# Complications of anemia in pregnancy: An updated overview for healthcare professionals

**DOI:** 10.1097/MD.0000000000044246

**Published:** 2025-08-29

**Authors:** Getrude Uzoma Obeagu, Emmanuel Ifeanyi Obeagu

**Affiliations:** a Department of Nursing Education, School of Nursing Science, Kampala International University, Ishaka, Uganda; b Department of Biomedical and Laboratory Science, Africa University, Mutare, Zimbabwe.

**Keywords:** anemia, fetal health, healthcare interventions, hemoglobin, iron-deficiency, maternal health, pregnancy

## Abstract

Anemia in pregnancy is a critical public health concern, affecting millions of women globally, particularly in low-resource settings. Defined by hemoglobin levels below 11 g/dL, this condition is primarily caused by nutritional deficiencies, chronic diseases, and genetic disorders, leading to severe maternal and fetal complications. This review provides an updated and comprehensive overview of the complications of anemia in pregnancy, highlighting the importance of early detection, effective management, and preventive strategies to mitigate its adverse outcomes. Maternal complications of anemia during pregnancy include an increased risk of mortality, susceptibility to infections, preterm labor, postpartum hemorrhage, and cardiac issues. Fetal complications are equally concerning, with risks of intrauterine growth restriction, preterm birth, perinatal mortality, and long-term neurodevelopmental impairments. Understanding these complications underscores the necessity for routine screening, accurate diagnosis, and timely intervention to improve health outcomes for both mother and child. Effective management of anemia in pregnancy involves a multifaceted approach, including nutritional supplementation, dietary modifications, and medical interventions such as parenteral iron therapy and blood transfusions. Prevention through regular antenatal care, health education, and public health initiatives is essential. By equipping healthcare professionals with the knowledge and tools to address anemia in pregnancy, this review aims to enhance maternal and fetal health outcomes, ultimately reducing the global burden of this condition.

## 1. Introduction

Anemia during pregnancy is a significant public health issue that affects millions of women worldwide, with a particularly high prevalence in low-resource settings. The condition is often underdiagnosed and undertreated, leading to a range of adverse maternal and fetal outcomes.^[1,2]^ The global burden of anemia in pregnancy is considerable, with estimates suggesting that approximately 41.8% of pregnant women worldwide are affected. The prevalence is particularly high in developing countries, where factors such as poor nutrition, limited access to healthcare, and high rates of infectious diseases contribute to the high incidence of anemia. Despite significant advancements in medical science and public health interventions, anemia in pregnancy remains a persistent challenge, highlighting the need for continued research and targeted interventions to address this issue.^[3,4]^ Iron-deficiency anemia is the most common type of anemia during pregnancy, accounting for the majority of cases. This condition arises from inadequate iron intake, poor absorption, or increased iron requirements during pregnancy. Folate deficiency and vitamin B12 deficiency are also significant contributors to anemia in pregnant women. Additionally, hemolytic anemias, caused by genetic disorders such as sickle cell disease and thalassemia, pose unique challenges in the management of anemia during pregnancy.^[5,6]^ The physiological changes that occur during pregnancy, including increased blood volume and erythropoiesis, further complicate the management of anemia. These changes result in hemodilution, which can mask the severity of anemia and delay diagnosis.^[7,8]^

Anemia during pregnancy is associated with a range of maternal complications that can have severe consequences. These include an increased risk of maternal mortality, heightened susceptibility to infections, preterm labor, preterm premature rupture of membranes, postpartum hemorrhage, and cardiac complications. Each of these complications can significantly impact the health and well-being of the mother, underscoring the importance of early detection and effective management of anemia in pregnant women.^[9,10]^ Fetal complications arising from maternal anemia are equally concerning. Intrauterine growth restriction (IUGR), preterm birth, perinatal mortality, and long-term neurodevelopmental impairments are some of the adverse outcomes associated with anemia in pregnancy. The reduced oxygen-carrying capacity of the blood in anemic mothers can lead to inadequate oxygen supply to the fetus, affecting fetal growth and development. These complications highlight the critical need for comprehensive prenatal care and targeted interventions to address anemia during pregnancy.^[4,11]^ The diagnosis of anemia in pregnancy involves a combination of clinical assessment and laboratory investigations. Routine blood tests, including complete blood count, serum ferritin levels, peripheral blood smear, and reticulocyte count, are essential for accurate diagnosis and assessment of anemia severity. Identifying the underlying cause of anemia is crucial for developing an effective treatment plan, which may include nutritional supplementation, dietary modifications, parenteral iron therapy, blood transfusion, and treatment of underlying conditions.^[12–14]^ Effective management of anemia during pregnancy requires a multifaceted approach that includes prevention, early diagnosis, and appropriate treatment. Nutritional supplementation with iron and folic acid is a cornerstone of anemia prevention and management. In severe cases or when oral supplementation is not effective, intravenous iron therapy or blood transfusions may be necessary. Addressing the underlying causes of anemia, such as infections or chronic diseases, is also critical to improving maternal and fetal outcomes.^[15]^ Prevention of anemia in pregnancy involves a combination of public health strategies and individual interventions. Regular antenatal care visits provide opportunities for early detection and management of anemia, while health education programs can raise awareness about the importance of nutrition and prenatal vitamins. Food fortification programs and initiatives to improve access to healthcare in low-resource settings are also essential components of anemia prevention.^[16]^

## 2. Aim

The aim of this review article is to provide an updated and comprehensive overview of the complications associated with anemia in pregnancy for healthcare professionals.

## 3. Rationale

Anemia during pregnancy remains a pervasive and critical public health issue, especially in low-resource settings where it contributes significantly to maternal and fetal morbidity and mortality. Despite advances in medical care and public health initiatives, the prevalence of anemia in pregnancy remains high, underscoring the need for continued attention and action. Anemia in pregnancy affects approximately 41.8% of pregnant women worldwide, with a disproportionate impact on women in developing countries. The causes of anemia in pregnancy are varied, including nutritional deficiencies (iron, folate, vitamin B12), chronic diseases, genetic hemoglobinopathies, and infections. A comprehensive understanding of these etiologies is crucial for accurate diagnosis and targeted treatment. Anemia during pregnancy is associated with severe maternal complications such as increased mortality risk, susceptibility to infections, preterm labor, postpartum hemorrhage, and cardiac issues. Addressing these complications through timely intervention can save lives and improve health outcomes. The impact of maternal anemia on fetal health is profound, leading to intrauterine growth restriction, preterm birth, perinatal mortality, and long-term neurodevelopmental impairments. Ensuring adequate maternal hemoglobin levels is essential for optimal fetal development. Healthcare professionals must stay informed about the latest evidence-based practices to provide the best care. Effective management of anemia in pregnancy requires a combination of nutritional supplementation, dietary modifications, parenteral iron therapy, and, in severe cases, blood transfusion.^[17]^

## 4. Literature search

A thorough literature search was conducted using electronic databases including PubMed, Google Scholar, and Cochrane Library. The search was designed to identify studies published in English from 2000 to 2023. The keywords used in the search included “anemia in pregnancy,” “maternal complications,” “fetal complications,” “iron-deficiency anemia,” “nutritional deficiencies,” “hemoglobinopathies,” “management of anemia,” and “prevention of anemia.”

The search strategy was aimed at capturing a wide range of studies, including randomized controlled trials, cohort studies, case-control studies, systematic reviews, and meta-analyses. Additional articles were identified through manual searches of the references cited in key publications.

## 5. Selection of relevant studies

The initial search yielded a large number of articles, which were then screened for relevance based on their titles and abstracts. Inclusion criteria were studies that focused on anemia in pregnancy, its complications, and management strategies. Exclusion criteria included studies that were not peer-reviewed, studies with nonpregnant populations, and articles published in languages other than English.

A full-text review was conducted on the articles that met the inclusion criteria. During this phase, studies that provided detailed information on the etiology, pathophysiology, clinical manifestations, maternal and fetal complications, diagnostic methods, and management strategies of anemia in pregnancy were prioritized.

## 6. Data extraction

Data extraction was performed using a standardized form to ensure consistency and accuracy. The extracted data included the study design, population characteristics, type of anemia, diagnostic criteria, complications reported, management strategies, and outcomes. Key findings and relevant statistics from each study were recorded.

## 7. Anemia in pregnancy

The prevalence of anemia in pregnancy varies widely, with the highest rates observed in developing countries. This condition arises from various causes, including nutritional deficiencies, chronic diseases, infections, and genetic disorders, making it a complex issue to address. The most common type of anemia during pregnancy is iron-deficiency anemia, which accounts for the majority of cases. This type of anemia occurs due to inadequate dietary intake of iron, poor absorption of iron from the gastrointestinal tract, or increased iron requirements during pregnancy. Other significant types include megaloblastic anemia, primarily due to folate or vitamin B12 deficiency, and hemolytic anemias caused by genetic conditions such as sickle cell disease and thalassemia. These different etiologies require tailored diagnostic and management strategies to effectively treat and prevent anemia in pregnant women. Anemia during pregnancy is associated with a range of complications that can adversely affect both the mother and the fetus. Maternal complications include an increased risk of mortality, heightened susceptibility to infections, preterm labor, postpartum hemorrhage, and cardiac issues. Fetal complications are equally concerning, with risks of intrauterine growth restriction, preterm birth, perinatal mortality, and long-term neurodevelopmental impairments. The reduced oxygen-carrying capacity of anemic blood can lead to insufficient oxygen delivery to the fetus, impacting its growth and development. Early detection, appropriate management, and preventive measures are essential to mitigate these risks and improve health outcomes for both mothers and their babies.^[18,19]^

Haemoglobin is a major component of red blood cells. It has strong affinity for oxygen and binds with oxygen to form oxy- hemoglobin. Iron is a major component of hemoglobin and functions mainly in Oxygen transport by hemoglobin. It is routinely absorbed by immature red blood cells through transferrin cycle. Plasma transferrin binds iron to enter cells through membrane-bound transferrin receptors to be used in haem biosynthesis.^[19]^ According to Klinken hemoglobin production begins in Proerythroblasts, which is a large cell with a large nucleus and small basophilic cytoplasm due to a high concentration of ribosomes.^[18]^ It gives blood its characteristics redness. Anemias are diagnosed on the basis of reduced red cell numbers, hemoglobin content and hematocrit (percentage of red cells in blood). Anemia in pregnancy is a condition characterized by a lower-than-normal level of red blood cells or hemoglobin in a woman’s blood during pregnancy. Hemoglobin is a protein in red blood cells that carries oxygen from the lungs to the rest of the body. Anemia can occur during pregnancy due to various factors, and it is a common concern for many expectant mothers.^[20]^ Also, in pregnancy, there is a physiological drop in hemoglobin (Hb) in the mid trimester.^[21]^ This physiological drop is attributed to increase of plasma volume and decrease of blood viscosity.^[22]^ Increase of plasma volume and decrease of blood viscosity led to better circulation in placenta.^[23]^

Anemia is relatively common during pregnancy. It is estimated that about 15% to 25% of pregnant women worldwide may experience anemia. During pregnancy, the volume of blood in the body increases to support the growing fetus. This dilution effect can lead to a decrease in the concentration of red blood cells. The most common cause of anemia in pregnancy is iron deficiency. Iron is essential for the production of hemoglobin. Inadequate iron intake or absorption can result in a decreased ability to produce enough hemoglobin.^[24–28]^ Anemia during pregnancy can lead to complications for both the mother and the developing fetus, including low birth weight, preterm birth, and developmental issues. Pregnant women are routinely screened for anemia during prenatal care through blood tests that measure hemoglobin levels. Additional tests may be conducted to identify the underlying cause of anemia. Iron supplementation is often prescribed to pregnant women with iron-deficiency anemia. Dietary changes to include iron-rich foods, such as lean meats, beans, leafy green vegetables, and fortified cereals.^[29–32]^ It is essential for pregnant women to attend regular prenatal checkups to monitor and address any signs of anemia. Early detection and appropriate management are crucial to ensure a healthy pregnancy for both the mother and the baby. Pregnant women should work closely with their healthcare providers to develop a personalized care plan based on their specific needs and circumstances.^[33,34]^

## 8. Complications of anemia in pregnancy

Severe anemia may have adverse effects on the mother and the fetus. Anemia with hemoglobin levels <6 g/d is associated with poor pregnancy outcome. Anemia in pregnancy can lead to increase in maternal and perinatal mortality, premature delivery, low birth weight, intra uterine growth retardation, infection, neurodevelopmental damage and bleeding complications.^[9]^ Perinatal mortality occur due to circulatory decompensation, elevated cardiac output, higher chance of hemorrhage with reduced compensatory mechanism for blood loss resulting to circulatory shock and death in severe anemia. Placental angiogenesis (development of new blood vessels) result from decreased levels of hemoglobin which limits oxygen supply to the fetus causing intrauterine growth restriction and fetal death. Interference with the normal red blood cell rheology and decreased alignments of platelets in the endothelial surface of blood cells due to reduction in red blood cell mass cause impaired hemostasis and hemorrhage associated with anemia in pregnancy.^[35]^ Also, Viana,^[36]^ reports that anemia associated with iron deficiency increases the risk of infection. Iron is needed for normal functioning of immunological systems like macrophages bactericidal activity of peroxide- and nitrous oxide-generating cellular enzyme and T-cell numbers and function. In accordance with Ali et al,^[37]^ severe anemia increases the risk of preeclampsia due to reduced bioavailability of micronutrients and antioxidants which is indicated by low blood levels of calcium, zinc and magnesium in pregnancy. Preeclampsia can occur due to vasoconstriction caused by free hemoglobin concentration. This is mainly seen in women with pregnancy induced hypertensions especially preeclampsia in which there is no increase in blood volume leading to an increase in hemoglobin concentration.^[38]^ In severe preeclampsia, there are reduced values of red blood cells, hemoglobin and hematocrit impairing oxygen delivery to mother and fetus.^[39]^ Prolonged hypoxia causes morphological and functional alterations in the placenta as well as adverse maternal and fetal outcome linked with preeclampsia syndrome and may lead to eclampsia.^[40]^ In addition, Sifakis, identified prematurity, spontaneous abortions, low birth weight, and fetal deaths as outcome of severe maternal anemia.^[41]^ Also, Debella et al,^[42]^ reported stillbirths, congenital deformities and premature birth occurred in 61.9% pregnant women with severe anemia. Moghaddam, states that low hemoglobin values during 3 trimesters of pregnancy were associated with low birth weight in fetus delivered by mothers who suffered anemia during pregnancy.^[43]^

Anemic mothers often experience fatigue and weakness, which can affect their ability to perform daily activities and may contribute to a decreased quality of life during pregnancy. Anemia can weaken the immune system, making pregnant women more susceptible to infections. Severe anemia in pregnancy has been associated with an increased risk of delivering a baby with low birth weight. Low birth weight is linked to an elevated risk of neonatal mortality and developmental issues. Anemic pregnant women may be at a higher risk of delivering prematurely, which can lead to complications for the baby, including respiratory distress syndrome and developmental challenges. Babies born to anemic mothers may have a higher risk of developing anemia themselves.^[38]^

Iron is crucial for the development of the baby’s brain. Severe iron-deficiency anemia during pregnancy may be associated with impaired cognitive development in the child. Women with iron-deficiency anemia may be at a higher risk of developing postpartum depression. Anemic women may be more prone to excessive bleeding during childbirth (postpartum hemorrhage), which can pose serious risks to both the mother and the baby. Anemia can put additional strain on the mother’s cardiovascular system, as the heart has to work harder to pump oxygen-depleted blood. Anemic pregnant women may have reduced exercise tolerance, affecting their ability to engage in physical activities.^[39]^ Table 1 shows complications associated with anemia in pregnancy

**Table 1:** Some of the potential complications associated with anemia in pregnancy.

Complication	Description
Maternal complications	
Fatigue and weakness	Anemic mothers often experience fatigue and weakness, affecting daily activities.
Increased risk of infections	Weakened immune system may make pregnant women more susceptible to infections.
Fetal complications	
Low birth weight	Severe anemia linked to an increased risk of delivering a baby with low birth weight.
Preterm birth	Anemic pregnant women may have a higher risk of delivering prematurely.
Neonatal anemia	Babies born to anemic mothers may have an increased risk of developing anemia.
Iron-deficiency anemia-specific complications	
Impaired cognitive development	Severe iron-deficiency anemia may be associated with impaired cognitive development.
Increased risk of postpartum depression	Iron-deficiency anemia may increase the risk of postpartum depression in mothers.
Increased blood loss during delivery	
Postpartum hemorrhage	Anemic women may be more prone to excessive bleeding during childbirth.
Cardiovascular strain on the mother	
Increased cardiovascular strain	Anemia can put additional strain on the mother’s cardiovascular system.
Impaired exercise tolerance	Anemic pregnant women may have reduced exercise tolerance.

## 9. Complications of anemia on fetus

Effect of anemia on fetus can be assessed by monitoring fetal well-being which reflects the physiologic status of the fetus in the uterus. Anemia in pregnancy leads to intrauterine growth restriction due to inadequate blood, oxygen and nutrient supply to the baby. Fetal distress occurs whenthe fetal heart tries to overcome the inadequate number or quality of red blood cells by pumping harder, manifesting as fetal tachycardia (increased fetal heart rate). It can result to fetal heart failure (hydrops fetalis) and intrauterine fetal death.^[44]^

Anemia in pregnancy caused by inadequacy of iron leads to changes in the energy metabolism in the brain with impaired neurotransmitter function and myelination resulting in impaired cognitive development.^[44]^ It is also associated with the release of corticotrophin-releasing hormone into maternal and fetal circulation from placenta. An increased level of corticotrophin-releasing hormone initiates preterm birth.^[45]^ Anemia causes retardation of fetal growth due to decreased blood flow, oxygen and nutrient delivery to placenta.^[46]^ Anemia is associated with low haemoglobin levels and its resultant effect are low Apgar score, compromised birth weight, small for gestational age babies, preterm labor, intrauterine growth retardation or intrauterine death. Early recognition and proper treatment help to prevent adverse effect associated with anemia in pregnancy.^[47]^ Table 2 shows complications of anemia on the fetus during pregnancy

**Table 2:** Complications of anemia on the fetus during pregnancy.

Complication	Description
Low birth weight	Severe anemia in the mother is associated with an increased risk of delivering a baby with low birth weight.
Preterm birth	Anemic pregnant women may have a higher risk of delivering prematurely, leading to potential complications for the baby.
Neonatal anemia	Babies born to anemic mothers may have an increased risk of developing anemia themselves.
Impaired cognitive development	Severe iron-deficiency anemia during pregnancy may be associated with impaired cognitive development in the child.
Respiratory distress syndrome	Preterm birth associated with maternal anemia can increase the risk of respiratory distress syndrome in the newborn.
Developmental challenges	Low birth weight and preterm birth linked to anemia may contribute to developmental challenges for the baby.
Increased risk of infections	Anemia in the mother can weaken the fetal immune system, potentially increasing the risk of infections in the newborn.

## 10. Recommendations

Implement universal screening for anemia during prenatal visits to identify cases early. This can involve assessing hemoglobin levels and, if possible, additional markers of iron deficiency to enable timely interventions. Provide comprehensive nutritional education to pregnant individuals, emphasizing iron-rich foods and the importance of iron supplementation when needed. Tailor iron supplementation based on individual needs and tolerability. Develop individualized treatment plans based on the severity of anemia and the patient’s health status. Consider factors such as comorbidities, diet, lifestyle, and potential medication interactions. Ensure consistent monitoring of hemoglobin levels throughout pregnancy and postpartum. Follow-up appointments should be scheduled to assess the response to treatment and adjust interventions as necessary.

Encourage multidisciplinary collaboration among obstetricians, hematologists, nutritionists, and other relevant healthcare professionals to provide comprehensive care for pregnant individuals with anemia. Educate pregnant individuals about the risks associated with anemia in pregnancy, emphasizing the importance of adherence to treatment plans and regular follow-ups. Provide adequate support and resources to facilitate compliance. Investigate and address underlying causes contributing to anemia in pregnancy, which may include nutritional deficiencies, chronic diseases, or other medical conditions. Continue monitoring and addressing anemia in the postpartum period. Follow-up appointments should be scheduled to assess the restoration of hemoglobin levels and manage any ongoing issues. Support public health initiatives aimed at raising awareness about anemia in pregnancy, emphasizing preventive measures, adequate nutrition, and access to prenatal care for all pregnant individuals. Encourage and support research efforts to better understand the nuances of anemia in pregnancy, its complications, and the effectiveness of different interventions to continually improve management strategies.

## 11. Conclusion

Anemia in pregnancy is a significant public health issue that poses serious risks to both maternal and fetal health. The high prevalence of anemia during pregnancy, particularly in low-resource settings, underscores the urgent need for effective interventions. Iron-deficiency anemia remains the most common form, but deficiencies in folate and vitamin B12, along with hemolytic anemias, contribute significantly to the overall burden. Maternal complications of anemia during pregnancy, such as increased mortality risk, susceptibility to infections, preterm labor, postpartum hemorrhage, and cardiac issues, emphasize the critical importance of early detection and appropriate management. Similarly, the adverse fetal outcomes, including intrauterine growth restriction, preterm birth, perinatal mortality, and long-term neurodevelopmental impairments, highlight the need for comprehensive prenatal care and intervention. Diagnostic approaches involving routine blood tests and early screening are essential for identifying and managing anemia in pregnancy. Effective management strategies include nutritional supplementation, dietary modifications, parenteral iron therapy, and, in severe cases, blood transfusion. Addressing the underlying causes of anemia is also vital for improving health outcomes. Prevention of anemia in pregnancy should be a priority, involving regular antenatal care, health education, and public health initiatives such as food fortification programs. These measures can significantly reduce the incidence and severity of anemia, leading to better maternal and fetal health outcomes.

## Author contributions

**Conceptualization:** Getrude Uzoma Obeagu.

**Methodology:** Getrude Uzoma Obeagu, Emmanuel Ifeanyi Obeagu.

**Supervision:** Emmanuel Ifeanyi Obeagu.

**Visualization:** Emmanuel Ifeanyi Obeagu.

**Writing – original draft:** Getrude Uzoma Obeagu, Emmanuel Ifeanyi Obeagu.

**Writing – review & editing:** Getrude Uzoma Obeagu, Emmanuel Ifeanyi Obeagu.
